# The Ribosome Biogenesis—Cancer Connection

**DOI:** 10.3390/cells8010055

**Published:** 2019-01-15

**Authors:** Marianna Penzo, Lorenzo Montanaro, Davide Treré, Massimo Derenzini

**Affiliations:** 1 Department of Experimental, Diagnostic and Specialty Medicine, Alma Mater Studiorum-University of Bologna, 40138 Bologna, Italy; marianna.penzo@unibo.it (M.P.); lorenzo.montanaro@unibo.it (L.M.); davide.trere@unibo.it (D.T.); 2 Center for Applied Biomedical Research (CRBA), Alma Mater Studiorum-University of Bologna, 40138 Bologna, Italy; 3 Alma Mater Studiorum-University of Bologna, 40126 Bologna, Italy

**Keywords:** nucleolus, ribosome biogenesis, ribosomal proteins, p53, AgNORs, tumour pathology, chronic inflammatory diseases, ribosomopathies, tumourigenesis, cancer chemotherapy

## Abstract

Multifaceted relations link ribosome biogenesis to cancer. Ribosome biogenesis takes place in the nucleolus. Clarifying the mechanisms involved in this nucleolar function and its relationship with cell proliferation: (1) allowed the understanding of the reasons for the nucleolar changes in cancer cells and their exploitation in tumor pathology, (2) defined the importance of the inhibition of ribosome biogenesis in cancer chemotherapy and (3) focused the attention on alterations of ribosome biogenesis in the pathogenesis of cancer. This review summarizes the research milestones regarding these relevant relationships between ribosome biogenesis and cancer. The structure and function of the nucleolus will also be briefly described.

## 1. Introduction

The nucleolus is the site where ribosomal genes are located during the interphase and where ribosome biogenesis occurs. In mammalian cells, ribosomal genes are transcribed by RNA polymerase I (Pol I) to produce the 47S rRNA precursor. At least three basal factors, termed transcription initiation factor I (TIF-I) A, selectivity factor 1 (SL1), and upstream binding factor (UBF), are necessary for ribosomal DNA (rDNA) transcription. The rRNA transcript undergoes site-specific modifications (including ribose methylation, pseudouridylation, base methylation and acetylation) and processing to generate mature 18S, 5.8S, and 28S rRNA. 5S rRNA is transcribed in the nucleoplasm by RNA polymerase III (Pol III) and also imported to the nucleolus [1,2]. During ribosome biogenesis, rRNAs are assembled with ribosomal proteins (RPs) to produce, in the end, mature ribosomal subunits.

**Figure 1:**
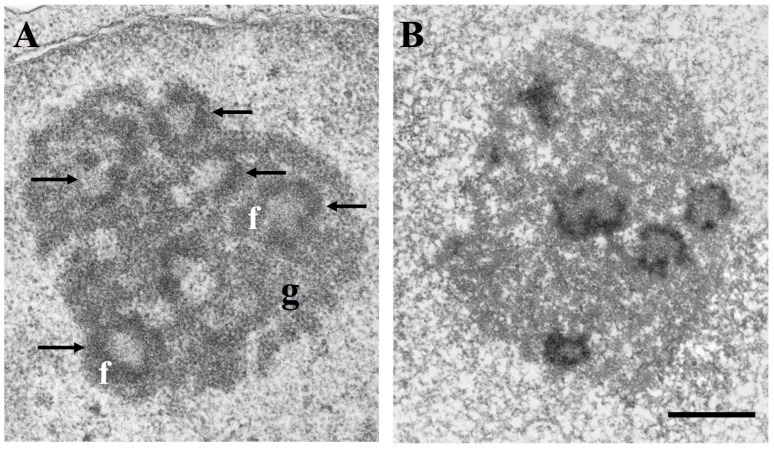
Nucleolar ultrastructure and location of the silver-stained Nucleolar Organizer Region (NOR) proteins in the fibrillar components; (**A**) electron microscope visualization of a nucleolus of a TG cell (established cell line from a human fallopian tube cancer) after uranium and lead staining. Arrows indicate fibrillar centres with the peripheral rim of dense fibrillar component (f). The granular component (g) surrounds the fibrillar components. Bar = 0.5 µm; (**B**) Electron microscope visualization of a TG cell nucleolus after specific silver staining for NOR proteins. Only the fibrillar centres and the associated dense fibrillar component are stained by silver. Bar = 0.5 µm.

At the ultrastructural level, three distinct morphological components are constantly present in the nucleolus: the fibrillar centres, light electron-dense roundish structures varying in size and number, the dense fibrillar component constituted by densely packed fibrils located at the border of fibrillar centres, and the granular component made of granules surrounding the fibrillar components (Figure 1A). Ribosomal genes are present in the fibrillar components together with all factors necessary for rDNA transcription, and the newly synthesized rRNA molecules are located in the dense fibrillar component. Therefore, the fibrillar components represent the structural-functional units of the nucleolus [3,4,5,6,7]. The granular component contains the rRNAs assembled with the ribosomal proteins (RPs) to give rise to the small 40S and large 60S subunits. The small 40S subunit contains one 18S rRNA and 33 ribosomal proteins (RPS), whereas the large 60S subunit contains one each of the 28S, 5.8S, and 5S rRNAs, together with 47 ribosomal proteins (RPL). The 60S and 40S subunits migrate from the granular component of the nucleolus to the cytoplasm where they constitute the 80S ribosome particle [1,2]. Some of the proteins present in the fibrillar components of the nucleolus—such as the largest RNA polymerase I subunit, UBF and nucleolin—are selectively stained by a silver (Ag) staining method that is used to visualize the nucleolar organizer regions (NORs) on metaphase chromosomes. Therefore, these proteins are known as AgNOR proteins and the silver-stained fibrillar components of the nucleolus are called silver-stained (Ag) interphase NORs, or more simply, AgNORs (Figure 1B) [8,9,10].

A wide range of data has revealed the fundamental importance of the nucleolus, of its functions in ribosomal biogenesis, and of its relationship with cell proliferation in many aspects of neoplastic disease. Here we review the most relevant research data which clarified the diagnostic meaning of the changes of the nucleolar size in tumor pathology, formed a rational basis for a new therapeutic approach to cancer based on ribosome biogenesis inhibitors and indicated a possible role of the nucleolar function in tumorigenesis.

The proteomic, genomic, and functional studies demonstrating that the nucleolus plays a role in the regulation of a series of mechanisms which are not related to ribosome biogenesis, and, nevertheless, once altered, might contribute to malignancy, are not the subject of the present work. For a thorough discussion of these data, the reader can refer to the exhaustive review by Lindström et al. [11].

## 2. The Nucleolus in Tumor Pathology

**Figure 2:**
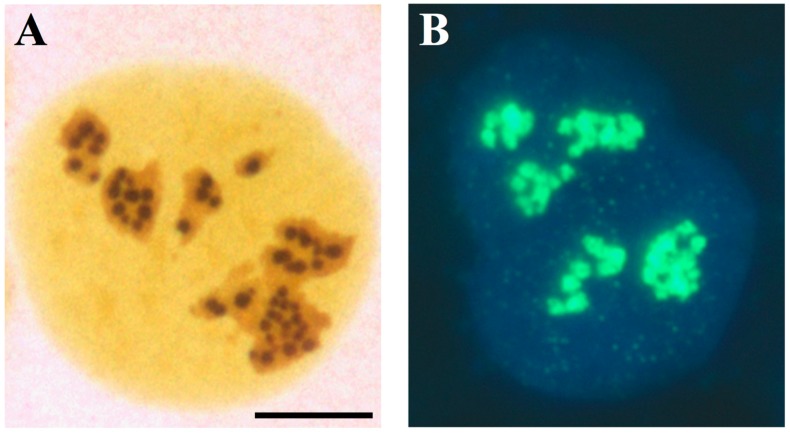
The AgNORs are the sites where rRNA synthesis takes place in the nucleolus; (**A**) light microscopy visualization of a U2OS cell (established cell line from a human osteosarcoma) after specific silver staining for NOR proteins. The silver-stained structures appear as well-defined, darkly stained dots clustered in the three nucleoli. (**B**) Visualization of rRNA synthesis in a U2OS cell, labelled with 5-fluorouridine (FUrd) for 15 min. FUrd was revealed by specific FITCH-conjugated monoclonal antibodies. DAPI counter-staining. Note the spot-like distribution of the sites of the nucleolar rRNA transcription, which overlaps that of the silver-stained dots in Figure 4. Bar = 10 µm.

Pathologists had focused on the nucleolus as a parameter for the diagnosis of tumour malignancy since the end of the nineteenth century [12], and hypertrophied nucleoli were considered a hallmark of cancer cells. However, whether the size of the nucleolus actually constituted a reliable parameter for distinguishing malignant from benign tumour cells remained undefined for a long time. In 1986, Ploton and colleagues [13] succeeded in visualizing the interphase AgNORs by light microscopy in routine paraffin sections by applying a modified, simple silver staining method for the NOR proteins. Using this method, the AgNORs appear as well-defined black dots distributed within the nucleolar body and perfectly identify the structural-functional units of the nucleolus (Figure 2A,B).

Since AgNOR distribution was found to be directly and strictly related to the size of the nucleolus and to its function in ribosome biogenesis [14,15], Ploton and colleagues’ silver-staining procedure represents a very useful tool to have information on the ribosome biogenesis rate in routinely processed histological sections, by either counting the AgNORs or by measuring the area occupied by the silver-stained structures with image analysis [16]. This achievement paved the way to studies aimed at defining the importance of alterations in nucleolar size and functional activity and their significance in tumor pathology. The systematic application of Ploton et al.’s silver staining method [13] on histological sections of almost every type of solid and hematological tumor clarified the importance of nucleolar alterations in the diagnosis of neoplasia and how these changes are related to the altered biological functions of neoplastic cells.

This series of studies concluded that malignant tumors generally have larger nucleoli than corresponding benign lesions of the same tissue. However, in many malignant tumors, such as breast cancer, thyroid cancer, cervical intraepithelial neoplasia, stomach cancer and endometrial lesions, cancer cells have nucleoli whose size overlaps that of the corresponding normal tissues and benign tumor lesions of the same histotype [17]. The absence of any nucleolar size overlap was demonstrated only between *naevocellular naevi* and melanocarcinomas and in pleural effusions between mesothelioma or metastatic cells and reactive cells. Therefore, with these exceptions, nucleolar size can help to distinguish a malignant from a benign tumor but cannot be generally considered to represent an absolute diagnostic parameter in tumor pathology [10,18,19,20].

Conversely, other studies, always conducted using the same silver staining procedure indicated that nucleolar size is directly related to the degree of cancer malignancy, and therefore represents a parameter predicting the clinical outcome of the disease [20]. Indeed, ribosome biogenesis, and hence nucleolar size, is conditioned by many of the characteristics acquired by cancer cells which may be expressed at different levels, even in tumors of the same histotype. Among these characteristics, the cancer growth rate (that is the percentage of proliferating cells) was found to be directly related to the mean nucleolar size of neoplastic cells [21]. The same was true for the doubling time of proliferating cells that was inversely related to nucleolar size and ribosome biogenesis rate [22]. Nucleolar size and these cell kinetics parameters are related because ribosome biogenesis increases in cycling cells [23] while in proliferating cells the shorter the cell cycle, the greater the ribosome biogenesis rate has to be in the time unit in order to reach a ribosome complement sufficient to give rise to normal daughter cells [24].

**Figure 3:**
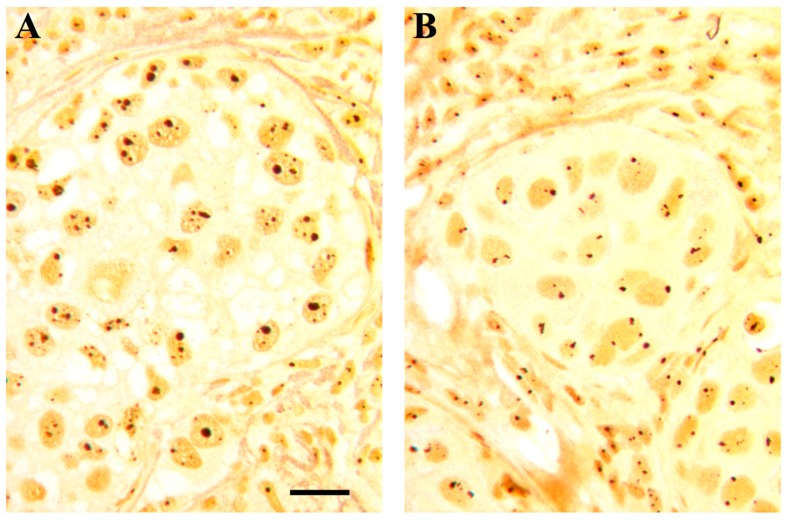
Histological sections from two cases of infiltrating ductal carcinomas after selective silver staining for NOR proteins; note the different quantitative distribution of the silver-stained structures in (**A**) (characterized by a high proliferation index, mutated p53 and RB loss) and (**B**) (characterized by a low proliferation index, wild type p53 and normal RB). Bar, 10 µm.

Other highly variable cancer cell characteristics influencing the function, and hence the size, of the nucleolus include the changes in the expression of oncogenes and tumor suppressor. For example, increased ribosome biogenesis rate may occur in some solid cancer and hematological malignancies as consequence of over expression of the oncogene *MYC*, which controls all the steps of ribosome biogenesis: it increases Pol I activity by facilitating the recruitment of SL1 to promoters, stimulates ribosomal protein synthesis by increasing Pol II transcription, and enhances Pol III transcription by activating Transcription Factor for polymerase III B (TFIIIB) [25,26,27]. Regarding the tumor suppressors, beyond the fact that rRNA transcription may be stimulated in some types of cancer by deletion or mutation of PTEN which normally represses Pol I transcription by disrupting the SL1 complex [28], there is evidence that the usual increase of ribosome biogenesis in cancer cells is the consequence of the very frequent alterations of the status of the two major tumor suppressors, TP53 and RB. p53 and pRb not only negatively control cell proliferation, but also hinder the transcription of ribosomal genes [29]. p53 and pRb frequently lose their function in human cancers, causing not only a loss of control of cell proliferation, but also the up-regulation of rRNA transcriptional activity. Therefore, tumors with altered p53 and/or pRb functions are characterized by a significantly larger nucleolus than tumors with normal pRb and p53 status [30,31] (Figure 3A,B). In conclusion, since a high growth rate, short doubling time, and loss of function of the two major tumor suppressors are the biological characteristics responsible for high cancer aggressiveness, it was no surprise that nucleolar size has been shown to be a valid prognostic parameter in neoplastic diseases [20].

Despite the very useful information that the quantitative analysis of the AgNORs can give regarding the cancer aggressiveness and the clinical outcome of the disease, the AgNOR staining reaction and the evaluation of the silver-stained nucleolar structures is still not officially recommended in tumor pathology. This may be due to economic reasons: in fact, the AgNOR staining reaction is difficult to be automatized, thus necessitating dedicated laboratory technicians.

## 3. Ribosome Biogenesis and Cancer Chemotherapy

### 3.1. Ribosome Biogenesis and Cell Cycle Regulation

Studies on the role of the nucleolus in cancer chemotherapy stemmed from the work by Volarevic and colleagues [32], who demonstrated that impaired ribosome biogenesis induces a checkpoint control that prevents cell cycle progression. For the first time, it was shown that cell proliferation could be blocked by inhibiting the production of new ribosomes. Pestov and colleagues [33] subsequently demonstrated that the inhibition of cell proliferation by perturbed ribosome biogenesis was p53-dependent. The importance of p53 in the inhibition of cell proliferation upon hindered ribosome biogenesis was also later demonstrated in transgenic mice overexpressing Myc [34]. Reduction of ribosome biogenesis by haploinsufficiency of either RPL24-uL24 or RPL38-uL38 decreases cell proliferation and delays tumor formation in p53 wild-type but not in p53 null mice. Ribosome biogenesis perturbation hinders cell proliferation by blocking the G_1_/S phase transition through the p21-mediated inhibition of pRb phosphorylation [33]. In fact, in cycling cells, the passage through the G_1_/S phase restriction point is controlled by the retinoblastoma tumor suppressor protein (pRb), which interacts with the E2Fs family of transcription regulators. E2Fs regulate the expression of those genes whose products are necessary for S phase progression. In its hypo-phosphorylated form, pRb is bound to E2Fs, thereby preventing these factors activating the E2Fs target genes; in its hyperphosphorylated form, pRb leaves the E2Fs free to activate the target genes. pRb phosphorylation is initiated by cyclin D-cyclin-Cdk-4 and -6 complexes in the early G_1_ phase and is completed by cyclin E-Cdk-2 complexes at the end of the G_1_ phase [35]. p53 stimulates the expression of p21Cip1 which inhibits the cyclin-dependent kinases thus hindering pRb phosphorylation [36] and triggering cell cycle arrest at G_1_ phase. Activation of the p53/p21/pRb pathway also triggers a G2 arrest sustained through an initial inhibition of cyclin B1-Cdc2, the cyclin-dependent kinase required to enter mitosis, followed by a marked decrease in cyclin B1 and Cdc2 levels [37,38].

The inhibition of ribosome biogenesis may induce cell cycle arrest also in a p53-independent manner. Specific inhibition of rRNA synthesis using the small-interfering RNA procedure to silence the *POLR1A* gene, which encodes the catalytic subunit of RNA polymerase I, hinders cell cycle progression in cells with inactivated p53, as a consequence of downregulation of the transcription factor E2F-1. Downregulation of E2F-1 is due to release of RPL11, which inactivated the E2F-1-stabilising function of the E3 ubiquitin protein ligase Mouse Double Minute 2 (MDM2) [39]. Reduction of cell proliferation was also found in p53-null cells after inhibition of ribosome biogenesis as consequence of RPL11-mediated downregulation of c-Myc activity. In fact, RPL11 binds to c-Myc, reducing its transcriptional activity and to c-Myc mRNA, promoting its degradation [40].

### 3.2. Ribosomal Stress and p53 Activation

Another major achievement was the elucidation of the molecular mechanisms underlying p53 activation upon ribosome biogenesis inhibition (see Figure 5 for schematic representation of the relationship between ribosome biogenesis rate and the level of p53 stabilization). The pioneering works in this field were those by Lohrum et al. [41], Zhang et al. [42] and Dai and Lu [43], who demonstrated that the p53 stabilization induced by inhibited rRNA synthesis was due to the fact that the ribosomal proteins L11-uL5 and L5-uL18, no longer used for ribosome building, bind to HDM2 thus preventing HDM2-mediated p53 ubiquitination and degradation. A series of other ribosomal proteins (RPS3-uS3, RPS7-eS7, RPS14-uS11, RPS15-uS19, RPS20-uS10, RPS25-eS25, RPS26-eS26, RPS27-eS27, RPS27a-eS31, RPL6-eL6, RPL23-uL14, RPS27L-eS27 like, RPL37-eL37) were subsequently shown to interact with HDM2 after inhibition of rRNA synthesis, thereby inducing p53 stabilization through the so-called RP-MDM2-p53 pathway (reviewed in [44,45,46,47]) to which RPL22-eL22 has recently been added [48]. Among the RPs binding to MDM2, RPL11-uL5 and RPL5-uL18 play a major role in MDM2 inactivation [41,42,43,49] by forming a complex with 5S rRNA, all the components of the complex being necessary for its inhibitory function [50,51].

### 3.3. Induction of Ribosomal Stress by Anticancer Agents

Rubbi and Milner [52] demonstrated the central role of impaired nucleolar function in determining p53 stabilization upon cellular stress, observing that major nuclear DNA damage failed to stabilize p53 unless the nucleolus was also disrupted. In other words, cellular damage of various kinds must also induce changes in nucleolar function in order to stabilize p53. Burger et al. [53] strengthened this concept by demonstrating that the alkylating and intercalating agents, antimetabolites, and topoisomerase and kinase inhibitors currently used for treating cancer also induce ribosome biogenesis inhibition, thus contributing to their toxic action on cancer cells. In this context it is worth noting that the alkylating agent oxaliplatin does not induce cancer cell death through DNA damage but through inhibition of ribosome biogenesis [54].

All these data have stimulated the development of new drugs designed to induce a selective inhibition of ribosomal biogenesis without the genotoxic effects typical of most currently used anticancer drugs. In this context, a small fluoroquinolone derivative (the CX-3543 molecule) was identified [55] and found to inhibit ribosome biogenesis by disrupting the interaction of rDNA G-quadruplexes with nucleolin, a nucleolar protein necessary for Pol I transcription [56]. Following this research line, another related compound, the CX-5461 molecule, was proposed as a selective inhibitor of rRNA transcription whose action reduces the binding affinity of the SL1 pre-initiation complex and RNA polymerase I complex to rDNA promoters [57,58]. Even though both molecules were highly effective in inhibiting ribosome biogenesis with cytostatic and cytotoxic effects on cancer cells, their mechanism of action also induced DNA damage [59]. CX-5461 is currently under clinical development (phase I studies). Another inhibitor of rRNA transcription, the BMH-21 molecule, is also a DNA intercalator which binds GC-rich sequences, of which ribosomal DNA genes are enriched, and potently represses RNA polymerase I (Pol I) transcription [60]. Unlike CX-5461 it lacks the common intercalator property of causing DNA damage [61].

Although this new approach to cancer chemotherapy is still in its infancy, the results so far obtained on the effects of inhibited ribosomal biogenesis on cancer cells should stimulate the development of new substances exclusively targeting nucleolar function. These inhibitors are particularly suited to cancer chemotherapy for two reasons: 1) they are not effective in resting cells due to the long half-life of cytoplasmic ribosomes [62,63], and 2) not only they can block cell cycle progression but also can induce the apoptotic death of neoplastic cells, especially those with a high ribosomal biogenesis rate [64]. Given their peculiar mechanism of action, the ribosomal biogenesis inhibitors can be associated with other currently used anticancer drugs which act through different cytotoxic pathways, thereby ensuring a more efficient destruction of cancer cells.

## 4. Ribosome Biogenesis and Neoplastic Transformation

It is well known that some human pathological conditions are associated with an increased risk to develop cancer. Among these, those which certainly have the most significant impact are the chronic inflammatory processes [65,66,67]. Also, a series of rare inherited disorders leading to the production of altered ribosomes (the so-called ribosomopathies), even though much less frequent than chronic infections, are characterized by a strong risk of cancer onset [68]. There is evidence that nucleolar functional changes play a role in tumor development in these two groups of human pathological conditions. More precisely, it was found that up-regulation of ribosome biogenesis rate is likely involved in neoplastic transformation of tissues affected by chronic inflammation, while ribosome dysfunctions are probably responsible for the increased cancer susceptibility in the above-mentioned ribosomopathies. It is worthwhile to point out that these alterations underlying cancer development only apparently contrast with each other. In fact, an unbalance in ribosome biogenesis rate (either an increase of rDNA transcription, or an alteration in the production of mature rRNAs or RPs) may ultimately lead, through different mechanisms, to the inactivation of p53.

How an up-regulated ribosome biogenesis and defects of ribosome biosynthesis may lead to cancer will be separately discussed.

### 4.1. Ribosome Biogenesis Rate and Cancer Development

**Figure 4:**
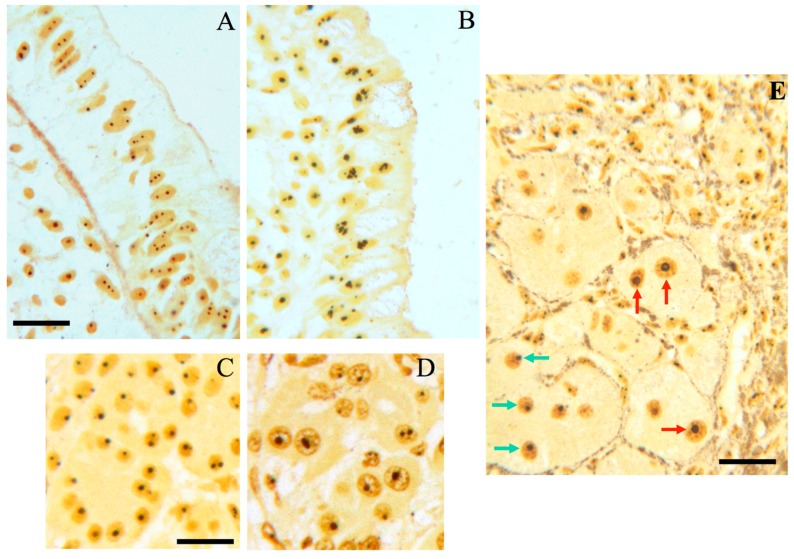
Chronic inflammatory diseases and nucleolar hypertrophy; (**A**) histological sections from normal human colon and (**B**) from human colon with ulcerative colitis (UC), after selective silver staining for NOR proteins. The size of nucleoli of the epithelial cells lining the surface of the normal mucosa is smaller than that of the epithelial cell nucleoli in UC. Bar, 10 µm. (**C**) Histological sections from normal human pancreas and (**D**) with chronic pancreatitis, after selective silver staining for NOR proteins (courtesy of Dr. M. Macchini, San Raffaele Scientific Institute, Department of Medical Oncology, Milan, Italy). Note the larger size of nucleoli of acinar cells in chronic pancreatitis than in normal pancreatic tissue. Bar, 10 µm; (**E**) histologic section from a human hepatitis B virus-related cirrhosis after selective silver staining for NOR proteins. Red arrows indicate hepatocytes with hypertrophic nucleoli, which are close to chronic inflammatory infiltrate. Green arrows indicate hepatocytes with normal-sized nucleoli. Bar, 10 µm.

Nucleolar hypertrophy, and therefore, an enhanced ribosome biogenesis rate, have been found in human hepatocytes of livers site of chronic inflammatory diseases [69], in epithelial cells of colon mucosa with chronic ulcerative disease [70], and in pancreatic acinar cells in patients with chronic pancreatitis [71] (Figure 4). All these conditions are characterized by an increased risk of cancer onset. Of note, in the case of chronic liver diseases, the higher the number of hepatocytes with nucleolar hypertrophy, the higher the rate of cancer occurrence [69].

**Figure 5:**
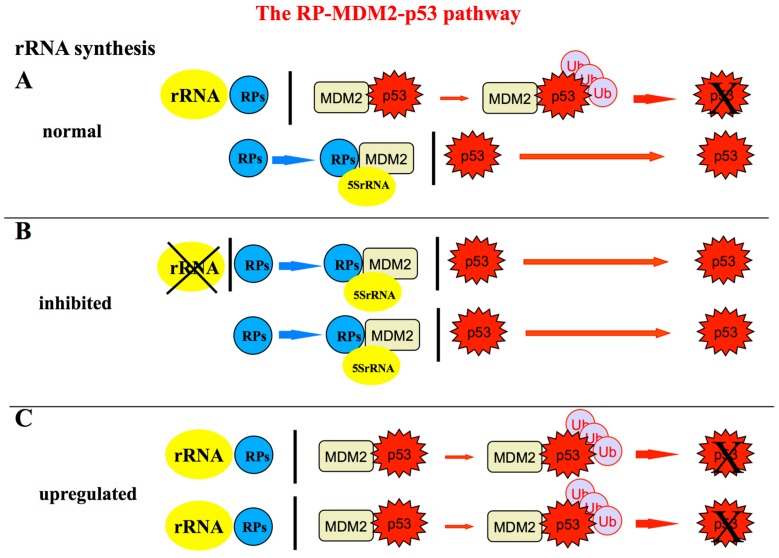
Schematic representation of the RPs-MDM2-p53 pathway showing the relationship between ribosome biogenesis rate and the level of p53 stabilization; (**A**) in normal conditions, the p53 level within the cell is maintained low because p53 is a short-lived protein that is rapidly degraded by murine double minute 2 (MDM2) and HDM2 in humans. In fact, MDM2 acts as an E3 ubiquitin ligase facilitating p53 proteasomal degradation [75,76,77]. Only a limited amount of MDM2 is inhibited by RPs not used for ribosome biogenesis. Among the RPs binding to MDM2, RPL11-uL5 and RPL5-uL18 play a major role in MDM2 inactivation [41,42,43,49] by forming a complex with 5S rRNA, all the components of the complex being necessary for its inhibitory function [50,51]; (**B**) perturbations of ribosome biogenesis (for example those caused by the treatment of rRNA transcription or processing inhibitors) are responsible for the fact that rRNAs are no longer available for ribosome construction. Then a greater amount of ribosomal proteins (RPs) is left free bind to MDM2 thus relieving its inhibitory activity toward p53 and allowing p53 to accumulate within the cell nucleus (reviewed in [44,47]); (**C**) On the contrary, when an up-regulation of rRNA synthesis occurs, a larger amount of RPs are used for ribosome building, being no longer available for MDM2 inactivation. Consequently, the level of p53 is reduced as result of a greater portion of MDM2 which is left free to induce p53 proteasomal degradation [74].

Regarding the causal relationship between chronic inflammation and nucleolar hypertrophic changes, it has been demonstrated that a major role is played by interleukin 6 (IL-6), which among the inflammatory substances released in the inflamed tissue, is that more strictly related to tumorigenesis [72,73]. Indeed, IL-6 stimulates c-MYC mRNA translation, which, in turn, is responsible for the upregulation of the ribosome biogenesis rate [70]. Regarding the link between an enhanced ribosome biogenesis and tumorigenesis, this is based on the fact that an up-regulation of rRNA transcription causes a lower availability of ribosomal proteins for MDM2 inactivation, being the utilization of the ribosomal proteins increased for the upregulated ribosome production. This leads to a higher downregulation of p53 protein expression as a consequence of an increased MDM2-mediated proteasomal degradation of the tumor suppressor [74] (see also Figure 5). The quantitative reduction of p53 is responsible for the acquisition of cellular phenotypic changes characteristic of epithelial-mesenchymal transition, such as a reduced level of E-cadherin expression, increased cell invasiveness and a decreased response to cytotoxic stresses both in transformed and untransformed human cell lines [70]. The same changes (nucleolar hypertrophy, reduced p53 expression and a focal reduction or absence of E-cadherin expression) were found in colon epithelial cells of patients with ulcerative colitis.

### 4.2. Qualitative Alterations in Ribosome Biogenesis and Cancer

Additional mechanisms through which altered ribosome biogenesis can lead to cancer have been ascribed to qualitative alterations in ribosome biogenesis. These can be very well appreciated when looking at a group of inherited disorders caused by mutation of genes whose products are involved in ribosome biogenesis with the consequent production of intrinsically altered ribosomes. These disorders are collectively defined as ribosomopathies and most of them share common clinical features including bone marrow failure, skeletal and skin abnormalities and, importantly, predisposition to cancer development [68,78]. Classically, ribosomopathies include Diamond-Blackfan Anemia (DBA), X-linked Dyskeratosis Congenita (X-DC), Cartilage-Hair Hypoplasia, Swachman-Diamond Syndrome and Treacher-Collins Syndrome (TCS). In addition to these better-defined disorders a growing number of additional diseases have been recently added to the list [68,79].

The link between ribosomopathies and cancer has been ascribed to two different, not necessarily mutually exclusive, major mechanisms. One mechanism may be related to the fact that altered ribosomes may translate differentially specific mRNAs ultimately increasing the expression of some oncogenes and reducing that of some tumor suppressors, such as p53 and p27 [80,81]. This has been demonstrated to happen in X-DC, where mutations in the DKC1 gene, encoding the pseudouridine synthase dyskerin, result in altered translation of the mRNAs encoding for the tumor suppressors p27 and p53 [82,83,84] and for a factor supporting tumor growth VEGF [85]. Importantly, all these translational alterations have been ascribed to an intrinsically altered activity of defectively pseudo-uridylated ribosomes [86]. In addition, also the reduction in the total amount of ribosome available for translation may induce specific dysregulation in protein synthesis [87].

**Figure 6:**
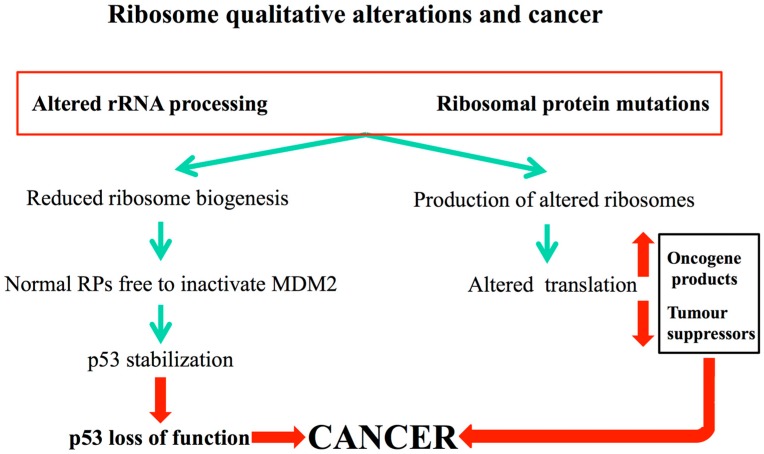
How qualitative alterations of ribosome biogenesis in ribosomopathies may lead to cancer; conditions qualitatively altering ribosome biogenesis include alterations in the rRNA modifying/processing factors and mutations in genes encoding for ribosomal proteins. These conditions (green arrows) can give rise both to a reduced ribosome biogenesis and to the formation of altered ribosomal subunits. Deregulated ribosome biogenesis activates the RP-MDM2-p53 pathway with consequent sustained p53 stabilization. Cells bypass this activation by p53 loss of function. The formation of altered ribosomes leads to unbalanced translation of tumour contrasting and promoting factors. The combined effect of the two different mechanisms would favor cancer onset (red arrows).

The second mechanism is related to the p53 response activation intrinsic to ribosomopathies. The reduction of rRNA production or the lack/mutations of specific ribosomal proteins occurring in ribosomopathies lead to the production of an excess of ribosomal proteins which are not incorporated into nascent ribosomes. These induce p53 stabilization by interacting with MDM2 (as above described). Activation of p53 has been indeed shown to be involved in failure of proliferating tissues observed in most ribosomopathies, as for example, DBA, TCS and X-DC [88,89,90,91,92]. In order to bypass the p53-mediated cell-cycle arrest, in some tissue the constant activation of ribosomal stress response may lead to a selective pressure for p53 loss of function. This may allow for cell survival, thus mitigating the effect on tissues specifically involved by ribosomopathies [93,94]. However, if such loss may have, on one side, a positive effect on specific features of the disorder, (e.g. bone marrow progenitors), on the other side it may increase patients’ cancer risk, by the selection of p53 compromised cells (see Figure 6).

Finally, the relationship between qualitative alterations of ribosome biogenesis and neoplastic transformation is also suggested by the frequent occurrence of ribosome changes in human sporadic cancer. Haploinsufficiency resulting from large deletions or truncating frameshift mutations has been reported in human cancers. *RPL5-uL18* inactivation has been described in 34% of breast cancers, in 28% of melanomas, in 11% of glioblastomas and in 2% of childhood T-cell Acute Lymphoblastic Leukemia (T-ALL) [95,96], while *RPL22-eL22* is mutated or is less expressed in several solid tumours (endometrial cancers, colorectal cancer, gastric cancer, breast carcinoma, and non-small cell lung carcinoma) [97,98,99,100]. Whether these ribosome changes may be responsible for neoplastic transformation has been not yet demonstrated. However, a reduced tumour suppressor activity of p53 can be reasonably postulated in those cases in which loss or mutation of the MDM2-inactivating RPs occur.

Furthermore, recent studies on paediatric cases of T-ALL suggest a driver role for RPL5-uL18 and RPL10-uL16 mutations in the development of this disease [101] and the De Keersmaecker group proposed also a p53-independent (ribosome-dependent?) mechanism underlying RPL5-uL18 inactivation–mediated cancer commitment in breast cancer and glioblastoma [95].

How qualitative changes of ribosomes might lead to cancer is schematically reported in Figure 6.

## 5. Concluding Remarks

The connection between altered ribosome biogenesis and cancer is now well established. Here, we revised the major achievements on the subject from different standpoints.

Alterations in nucleolar size, is recognized as valuable prognostic marker in many different kinds of tumours. In addition, the visualization of nucleolar patterns in routine histological analysis may give important hints on the prognosis of the malignancies, and possibly also valuable information on the advisability of ribosome-biogenesis targeting therapies. In particular, for those p53-competent tumours with nucleolar hypertrophy (and, therefore, with up-regulated ribosome biogenesis) a therapeutic approach selectively inhibiting rDNA transcription should be considered, evaluated on the basis of its clinical effect and possibly juxtaposed to other chemotherapies.

In light of the connection established between IL6-mediated stimulation of ribosome biogenesis in chronic inflammatory diseases and cancer, it will be worthy to define the role of increased ribosome biogenesis also in those metabolism-related diseases characterized by a persisting pro-inflammatory status with increased cancer onset, such as the insulin resistant states of obesity and type 2 diabetes. This would allow, in the long term, to identify preventive approaches to reduce cancer risk in these conditions. In this context it is worth noting that therapeutic dosage of aspirin downregulates ribosome biogenesis thus increasing the p53-mediated tumor-suppressor activity, being in this way able to reduce the risk of cancer onset, either or not linked to chronic inflammatory processes [102].

Finally, for those cancers where a qualitative ribosomal defect has been identified (i.e., ribosomes with altered structure due to RP mutations), further studies are needed to fully understand the contribution of these defects to tumour development. Only afterwards it will be possible to identify compounds and drugs specifically targeting structurally altered ribosomes, or pathways impaired downstream of ribosome biogenesis qualitative alterations.
